# Immunization Trials with Recombinant Major Sperm Protein of the Bovine Lungworm *Dictyocaulus viviparus*

**DOI:** 10.3390/pathogens11010055

**Published:** 2022-01-02

**Authors:** Andrea Springer, Christian von Holtum, Georg von Samson-Himmelstjerna, Christina Strube

**Affiliations:** Institute for Parasitology, Centre for Infection Medicine, University of Veterinary Medicine Hannover, 30559 Hannover, Germany; andrea.springer@tiho-hannover.de (A.S.); cvonholtum@gmx.de (C.v.H.); samson.georg@fu-berlin.de (G.v.S.-H.)

**Keywords:** major sperm protein, *Dictyocaulus viviparus*, recombinant vaccine, vaccination, immunization

## Abstract

The lungworm *Dictyocaulus viviparus* is one of the most economically important bovine parasites in temperate climate regions. Following infection, *D. viviparus* induces a temporary protective immunity, and a vaccine based on attenuated, infective larvae is commercially available. However, due to several disadvantages of the live vaccine, the development of a recombinant subunit vaccine is highly desirable. Therefore, the major sperm protein (MSP), which is essential for the parasite’s reproduction, was tested as a recombinantly *Escherichia coli*-expressed glutathione-S-transferase (GST)-fused vaccine antigen in immunization trials with two different adjuvants, Quil A and Al(OH)_3_. Calves (N = 4 per group) were immunized on study day (SD) 0, 21 and 42 and given a challenge infection on SD 63–65. The two control groups received only the respective adjuvant. Based on geometric means (GM), a 53.64% reduction in larvae per female worm was observed in the rMSP Quil A group vs. its control group (arithmetic means (AM): 54.43%), but this difference was not statistically significant. In the rMSP Al(OH)_3_ group, the mean number of larvae per female worm was even higher than in the respective control group (GM: 9.24%, AM: 14.14%). Furthermore, male and female worm burdens and the absolute number of larvae did not differ significantly, while the Al(OH)_3_ control group harbored significantly longer worms than the vaccinated group. Vaccinated animals showed a rise in rMSP-specific antibodies, particularly IgG and its subclass IgG1, and the native protein was detected by immunoblots. Although rMSP alone did not lead to significantly reduced worm fecundity, it might still prove useful as part of a multi-component vaccine.

## 1. Introduction

*Dictyocaulus viviparus*, the bovine lungworm, is one of the most economically important parasites in grazing cattle in temperate climate zones. It causes parasitic bronchopneumonia, which is often accompanied by severe clinical signs, and may even lead to death. Holzhauer et al. [1] estimated total costs of EUR 159 to 167 per cow due to lungworm outbreaks on two dairy farms in 2007. Furthermore, recent studies have shown that even subclinical lungworm infections may negatively affect production parameters such as milk yield, milk protein and milk fat content [2,3,4].

*Dictyocaulus viviparus* induces strong immune responses, which result in protection against clinical signs due to challenge infections for up to one year [5,6,7]. This protection is most likely antibody-mediated, as indicated by the experimental transfer of an immunoglobulin preparation from exposed to lungworm-naïve calves, which resulted in protection against challenge infection [8]. Consequently, lungworm control can be achieved—aside from by the regular use of anthelmintics and pasture management—by a live vaccine containing irradiated third-stage larvae (L3) (Bovilis^®^ Dictol or Bovilis^®^ Huskvac, respectively, MSD Animal Health). However, this vaccine is marketed in only a few European countries (e.g., the Netherlands, UK, Ireland and Switzerland), and needs to be imported for use in other EU member states. The vaccine induces protective immunity for one grazing season, but it has several disadvantages such as high production costs, a short shelf-life and the necessity of refrigerated storage. Furthermore, infected donor animals are needed to regularly produce larvae, raising ethical concerns [9]. These disadvantages have led to insufficient acceptance by veterinarians and farmers with the consequence of withdrawal from, for example, the German market.

In contrast to the live vaccine, a vaccine based on recombinant antigens could be produced cost-effectively in large amounts, with high purity and without the need for infected donor cattle. Additionally, lyophilization allows long-term storage without refrigeration. Consequently, biotechnologically manufactured antigenic protein vaccines could overcome the economical and ethical disadvantages of the live vaccine. Although the development of subunit vaccines against parasitic nematodes is challenging [10], vaccination with recombinant proteins or protein cocktails has yielded promising results against *Haemonchus contortus* [11] and *Teladorsagia circumcincta* [12,13] in sheep, for example. Regarding *D. viviparus*, vaccination trials using the recombinantly expressed muscle protein paramyosin as an antigen resulted in significant reductions of worm burden and larval shedding, although the magnitude of the effect was still inferior as compared to the live attenuated vaccine [14] and could not be reproduced in all trials [15]. Combined vaccination with additional recombinant antigens could further improve this promising approach [15]. 

In addition to paramyosin, the major sperm protein (MSP) appears as a promising vaccine candidate for the bovine lungworm. In fact, a patent for an immunogenic *D. viviparus* protein, putatively MSP, and its recombinant expression for immunodiagnosis and vaccination was already filed in 1997 [16]. This nematode-specific protein, which is almost exclusively transcribed in adult male lungworms [17], is essential for sperm motility and promotes oocyte maturation and ovulation [18,19]. Targeting MSP by vaccination might, therefore, significantly impair worm fecundity. Bovine lungworm MSP is highly antigenic in infected cattle and, consequently, an ELISA based on recombinant MSP (rMSP) has been developed for the immunodiagnosis of lungworm infections in serum and milk samples [20,21,22,23]. However, it is unknown whether the produced antibodies are protective as no vaccination trials have been published to date—if they were, inactivation of MSP by specific antibodies should result in strongly reduced larval offspring and, thus, considerably reduce pasture contamination. Consequently, the low numbers of larvae present on pasture would lead to low-level infections, contributing to the development of natural immunity without causing clinical signs. The present pilot study describes the use of bovine lungworm rMSP formulated with two different adjuvants (Quil A and Al(OH)_3_) in cattle immunization trials to test its potential as a recombinant subunit vaccine. 

## 2. Results

### 2.1. Condition of the Calves

Among the sixteen calves used in the study, no local reactions to vaccinations on study day (SD) 0, 21 and 42 were observed, except for one animal which showed slight local swelling for a few days after the first injection. No significant differences were observed between the vaccinated and their respective control groups in terms of weight gain (Mann–Whitney U test, U = 4, *p* = 0.34 and U = 6, *p* = 0.63, respectively). 

Following challenge infections with 1100 L3 on SD 63, 64 and 65 each, every study animal developed clinical dictyocaulosis. No differences in clinical signs, which included serous to mucopurulent nasal discharge, coughing, fever and dyspnea, could be observed between the control and vaccinated groups, as symptoms ranged from mild to severe in all four groups. Therefore, several animals had to be treated with antibiotics and non-steroidal anti-inflammatory drugs during the patent period of dictyocaulosis. Gross pathological examination of the lungs after necropsy confirmed multifocal mucopurulent bronchopneumonia in each animal with the proportion of affected lung tissue ranging from 5 to 90%. 

### 2.2. Parasitological Parameters (Worm Burden, Larvae Shedding and Worm Size) 

**Figure 1:**
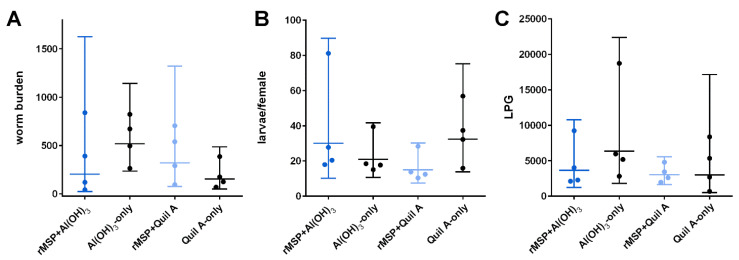
(**A**) Number of adult *D. viviparus* worms, (**B**) larvae per female worm and (**C**) sum of daily LPG values from SD 84–98 in vaccinated and control animals. Individual values of the calves are represented as dots. The horizontal lines indicate the geometric mean and the error bars 95% confidence intervals.

Parasitological parameters are summarized in Table 1. The number of adult worms showed large variation within the groups (Figure 1A). No significant differences in total worm burden were found between vaccinated and control groups (Mann–Whitney U test, U = 4, *p* = 0.34 and U = 5, *p* = 0.49, respectively). The female-to-male ratio was 1.6:1 to 1.8:1 in all groups. Differences in the number of larvae per female worm between vaccinated and adjuvant-only groups were not statistically significant (Mann–Whitney U test, U = 4, *p* = 0.34 and U = 1, *p* = 0.06, respectively, Figure 1B), although a reduction of 53.64% based on geometric means (GM; arithmetic means (AM): 54.43%) was observed in the Quil A group compared with the respective control group. However, in the rMSP Al(OH)_3_ group, the mean number of larvae per female worm was even higher than in the respective control group (Table 1). 

Based on GM, no reduction in larval shedding was observed in the rMSP Quil A group as compared to the Quil A-only control group (25.10% reduction based on AM, Figure 1C). In the rMSP Al(OH)_3_ group, a 42.55% reduction based on GM was observed as compared to the adjuvant-only group (AM: 46.12% reduction). However, differences between the vaccinated groups and the corresponding control groups were not statistically significant (Mann–Whitney U test, U = 4, *p* = 0.34 and U = 6, *p* = 0.66, respectively). 

**Figure 2:**
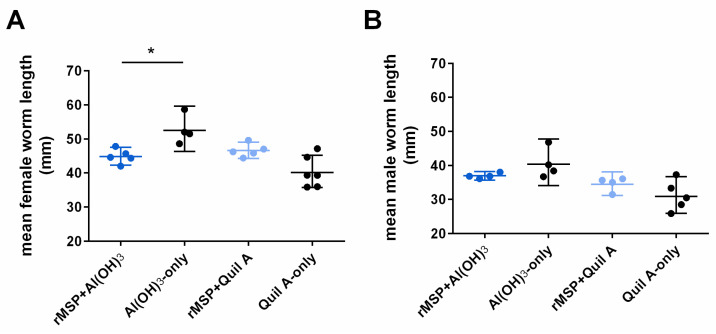
Length of (**A**) female and (**B**) male *D. viviparus* in vaccinated and control animals. Individual values of the calves are represented as dots. The horizontal lines indicate the geometric mean and the error bars 95% confidence intervals. The asterisk indicates a statistically significant difference (*p* < 0.05).

Regarding worm size, male worms did not differ in length between vaccinated and control groups (Mann–Whitney U test, U = 3, *p* = 0.20 and U = 5, *p* = 0.29). However, female worms were significantly shorter in the rMSP Al(OH)_3_ group than in the Al(OH)_3_-only control group (Mann–Whitney U test, U = 0, *p* = 0.02), whereas this was not the case when Quil A was used as adjuvant (U = 5, *p* = 0.08, Figure 2A).

### 2.3. Development of rMSP-Specific Antibodies

**Figure 3:**
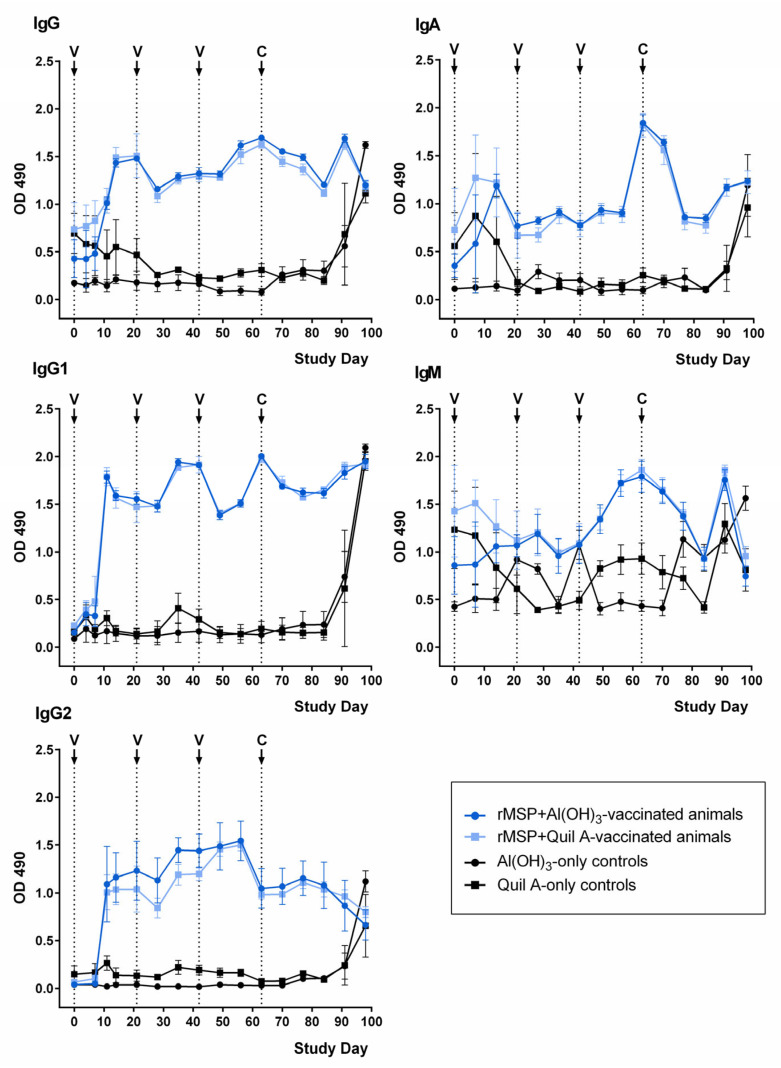
Anti-rMSP antibody development during vaccination trials. Mean optical density (OD) values are displayed over the course of the study for immunoglobulin (Ig) classes IgG, IgG1, IgG2, IgA and IgM for the different study groups. Error bars indicate standard deviations. V = vaccination, C = challenge infection.

Patterns of antibody development in the rMSP-vaccinated and control groups are presented in Figure 3. All rMSP-vaccinated animals developed specific antibodies in response to vaccination, and patterns were very similar in the rMSP Quil A and rMSP Al(OH)_3_ groups. 

The antibody level increase following the first rMSP vaccination was most remarkable for IgG, including both IgG1 and IgG2. These antibody (sub)classes subsequently showed an increase after each booster vaccination. After challenge infection, only IgG and IgG1 levels increased again. Animals in the control groups only showed an IgG increase in response to challenge infection.

IgA levels in the rMSP-vaccinated groups increased after the first vaccination; however, a similar peak also occurred in the Quil A control group before OD values in this group fell back to baseline. In contrast, OD values in the rMSP-vaccinated groups remained at a higher level and showed subsequent peaks after the second booster vaccination on day 42, as well as after challenge infection. Challenge infection also caused an IgA increase in the control animals. 

Regarding IgM, the pattern was less clear as optical density (OD) values fluctuated around the same level in the vaccinated and in the control groups. Only after the second booster vaccination on day 42 was a clear antibody level increase evident, which did not occur in the control groups. However, the levels declined again rapidly before showing a final rise due to challenge infection, which also occurred in the control groups.

### 2.4. Binding of Anti-rMSP Antibodies to Native MSP

**Figure 4:**
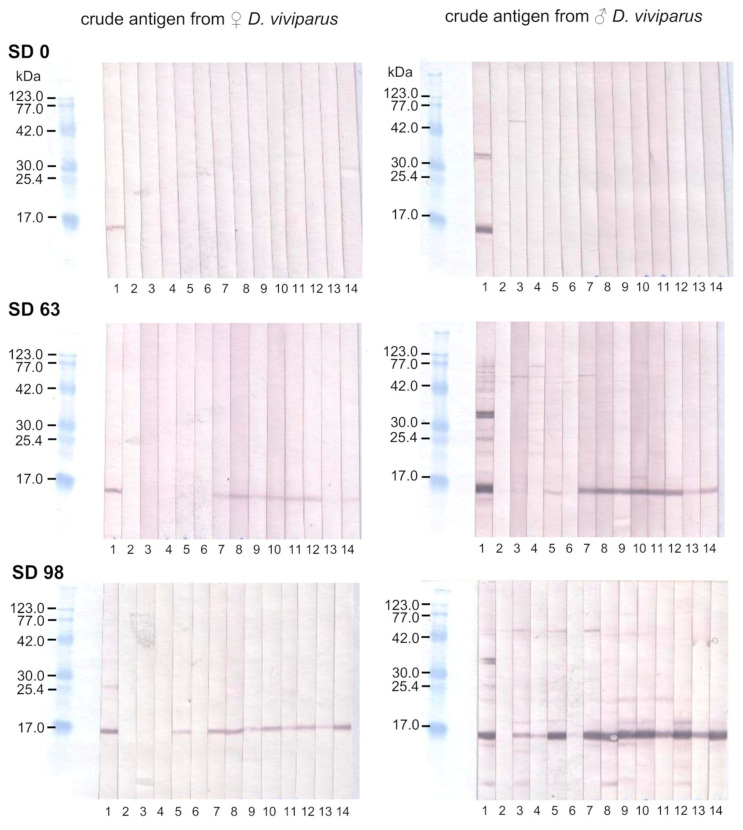
Immunoblot detection of MSP (approximately 14 kDa) in whole adult lungworm lysate of female (**left**) and male (**right**) worms by sera of the Quil-A-only control group (lanes 3–6), the rMSP Quil A group (lanes 7–10) and the rMSP Al(OH)_3_ group (lanes 10–14) from SD 0 (top), 63 (middle) and 98 (bottom). Lane 1: positive control serum, lane 2: negative control serum.

Immunoblot analysis showed binding of IgG antibodies from sera of vaccinated animals on SD 63 to an approximate 14 kDa fraction of crude lungworm antigen, corresponding to MSP [17], whereas there was no binding on SD 0 prior to vaccination (Figure 4). The intensity of the reaction was stronger with male than female crude lungworm antigens. One animal from the Quil A control group showed a band slightly below 14 kDa with a male, but not female, crude lungworm antigen on SD 63 (Figure 4, lane 5) prior to challenge infection. On SD 98, following challenge infection, animals of all three groups showed 14 kDa bands of varying intensity with a male crude lungworm antigen, although the serum of only one control animal produced a very weak band (Figure 4, lane 6).

**Figure 5:**
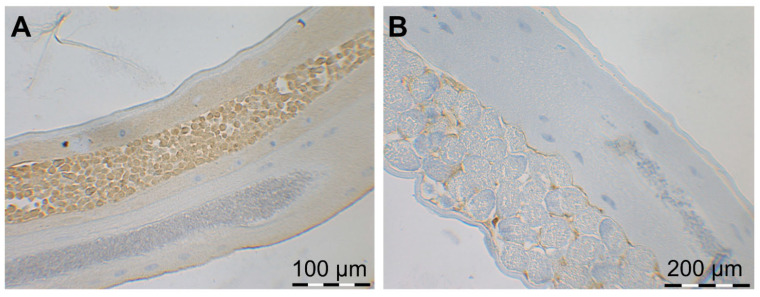
Immunohistochemical detection of MSP (dark orange staining) by a monoclonal anti-*C. elegans* MSP antibody in (**A**) the testes of a male and (**B**) surrounding the eggs in the uterus of a female *D. viviparus*.

To show the localization of native MSP in male and female worms, longitudinal sections of adult *D. viviparus* were analyzed by immunohistochemistry. Native MSP was detected by immunohistochemistry in the testes of adult male lungworms (Figure 5A) and surrounding the eggs in the uterus of adult female lungworms (Figure 5B). 

## 3. Discussion

At present, control of lungworm infections in cattle is mainly achieved via grazing management and strategic administration of anthelmintics. However, changing consumer awareness regarding metaphylactic treatment of livestock, as well as increasing resistance of parasites against anthelmintics, calls for the development of alternative control strategies. An effective live vaccine based on X-ray-attenuated L3 of *D. viviparus* has been available for decades [24]. However, limited shelf-life, as well as dependence on donor calves for the production of larvae, has led to insufficient acceptance of this vaccine [25] and its withdrawal from the market in several European countries. A subunit vaccine based on recombinant proteins could overcome these drawbacks [9]. In the present pilot study, recombinantly *E. coli*-expressed MSP of *D. viviparus* was evaluated as a vaccine candidate in cattle. Although a patent regarding isolation and recombinant expression of putative MSP for vaccination of cattle was already filed in 1997 [16], no vaccination trials have been published so far. As the present experiments were designed as a pilot study, only a limited number of animals was used. However, the obtained results do not warrant repeating the trial with a larger group size. Although the observed rise in antibody levels proved the antigenicity of rMSP, no statistically significant differences in parasite fecundity were demonstrated between rMSP-vaccinated and control groups due to large variation between animals. Moreover, vaccinated animals, as well as control animals, developed clinical dictyocaulosis upon challenge infection, and pathological examinations confirmed bronchopneumonia in all animals. 

As MSP is almost exclusively expressed in adult male lungworms [17], an effect on worm establishment and, thus, adult worm numbers and sex ratio was not necessarily expected. However, it was not clear if any effect on sperm development in males by anti-rMSP antibodies might also be detrimental for male development and survival. A significant difference in mean female worm length was detected between the rMSP Al(OH)_3_ group and the Al(OH)_3_-only control group; however, this was rather due to the fact that worms in the Al(OH)_3_-only control group were, on average, longer than in the other three groups. Therefore, it is unlikely that the observed difference was due to the rMSP vaccine. Likewise, Urwin et al. [26] showed that suppression of MSP expression by RNA interference had no effect on development of the plant-parasitic nematode *Heterodera glycines.*

However, MSP is essential for sperm motility, while also favoring oocyte maturation and ovulation [18,19]. Therefore, it was expected that effectively antagonizing MSP would result in considerably impaired worm fecundity, reducing pasture contamination with *Dictyocaulus* larvae. Impaired helminth fecundity was observed, for example, in vaccination trials with recombinant gut proteins of the canine hookworm, *Ancylostoma caninum* [27,28]. Herein, we observed a more than 50% reduced number of larvae per female following vaccination with rMSP in the Quil A group. However, these data were also not significantly different from the respective findings in the adjuvant-only control group. The poor effect might be due to several reasons. Apart from the small study size, further potential factors need to be considered in this concern. The selected expression system can be a crucial factor for successful immunization with recombinant proteins [29,30,31]. Proteins expressed by *E. coli,* as in this study, have no posttranslational modifications and may be incorrectly folded. However, it was expected that expression of rMSP in a prokaryotic system should have no disadvantages over expression in a eukaryotic system with regard to glycosylation, as MSPs are presumably secreted by a non-classical pathway which is independent of the ER–Golgi network and are, thus, most likely not glycosylated [17,32]. Indeed, analyses carried out by Hofmann and Schmid [16] indicated that native *D. viviparus* MSP is not glycosylated. 

Nevertheless, incorrect protein folding may have impaired vaccination success, al-though immunoblot analysis demonstrated that rMSP antibodies of vaccinated animals were able to bind to native MSP. On the one hand, binding may have been due to linear epitopes, which are unaffected by tertiary structure. On the other hand, the successful binding may indicate that the recombinant protein has at least been partially correctly folded, resulting in conformational epitopes identical to native MSP. Similarly, antisera raised against truncated regions of an effective *Echinococcus granulosus* vaccine antigen in sheep reacted with the native parasite protein but did not achieve the same level of protection as the complete antigen [33]. 

One animal from the Quil A-only control group showed a band at approximately 14 kDa in the immunoblot analysis prior to challenge infection (on SD 63). However, when tested by ELISA, the animal did not show elevated antibody levels at this study day. Furthermore, the band appeared to be slightly lower than the 14 kDa bands of the vaccinated animals and is, therefore, most likely attributable to nonspecific binding. Similar nonspecific bands just below the MSP band were visible in several animals on day 98. The mentioned control animal generally seemed to mount a rather strong immune response, as indicated by the strong MSP band after challenge infection (SD 98) in comparison to the weaker bands observed with sera of the remaining control animals. This was also supported by high anti-rMSP IgG and IgG1 levels in this animal on SD 91, resulting in the large standard deviation of IgG and IgG1 in the Quil A-only control group at this time point. The post-challenge serum of another control animal only showed a very weak reaction with crude male lungworm antigen in the immunoblot, although the IgG response, as measured by ELISA, did not differ from the remaining animals in this group. 

Furthermore, specific combinations of expression system and adjuvant may be crucial for vaccination success. For example, vaccination with *E. coli*-expressed recombinant paramyosin, a muscle protein, adjuvanted by Quil A considerably reduced *D. viviparus* burden and larvae shedding in one trial [14] while these results could not be repeated using *Pichia pastoris*-expressed paramyosin with different adjuvants [15]. In the present study, no significant influence of the two different adjuvants on vaccine efficacy was observed. Despite the fact that Quil A has the ability to stimulate a T helper cell 1 (Th1) reaction, whereas Al(OH)_3_ only causes a Th2 response [34], no differences between Quil A and Al(OH)_3_ regarding IgG, IgG1, IgG2, IgM and IgA levels were observed. Levels of IgG, including subclasses IgG1 and IgG2, as well as IgA, increased in all vaccinated animals and showed smaller peaks following booster vaccinations. In contrast, IgM values showed large fluctuations both in the vaccinated groups and in the control groups. The same was observed in previous *D. viviparus* vaccination trials [14,15,35] and might be due to copurified *E. coli* antigens accidentally present on the ELISA plates or an unspecific antibody binding to rMSP [15]. Due to the lack of a commercially available conjugate against bovine IgG2a, detection of IgG2a as a specific indicator of a Th1 response was not possible. In *D. viviparus* infections, a mixed Th1/Th2 response occurs, but a correlation with protection has only been observed regarding the Th2 arm of the immune system [36,37]. Similarly, vaccination experiments with larval antigens of *H. contortus* were only successful with Al(OH)_3_ as the Th1 response induced by Quil A rather exacerbated the infection [38,39]. Therefore, a stronger protection induced by the Al(OH)_3_-adjuvanted vaccine could have been expected. However, neither vaccination group showed a significant difference in parasitological parameters compared to the respective control group, and, although the average absolute number of larvae was reduced by more than 40% in the rMSP Al(OH)_3_ group, the number of larvae per female worm was even higher than in the respective control group. 

Finally, it remains possible that the MSP antibodies induced by vaccination were either non-protective antibodies or they might not have been able to effectively reach the antigen during infection. Although Scott et al. [40] showed an increase of IgG, as well as IgA, in broncho-alveolar lavage fluid (BALF) following *D. viviparus* infection, the patterns observed in BALF were not directly correlated to antibody levels in serum. Especially, IgG levels were lower in BALF than in serum, which may also be the case after vaccination. Therefore, a comparison of serum and BALF antibody levels should be considered in future vaccine trials. Furthermore, MSP fulfils its main functions in the parasite’s testes and—after being transferred from male to female during copulation—in the uterus and might not be easily accessible to antibodies in these organs in contrast to proteins located, e.g., in the parasite’s intestine or in muscle tissue, such as paramyosin [14].

Although rMSP alone did not lead to significantly reduced worm reproduction, it might still prove useful as part of a multi-component vaccine due to synergistic or additive effects. Recently, a multi-component vaccine based on recombinant proteins showed high levels of protection against *Teladorsagia circumcincta* in sheep [12,13], and this might be a valuable option to explore for *D. viviparus*.

## 4. Materials and Methods

### 4.1. Recombinant Expression in Escherichia coli

*Dictyocaulus viviparus* MSP was recombinantly expressed as a glutathione-S-transferase (GST)-fused protein in One Shot^®^ TOP 10 chemically competent *E. coli* using the expression vector pGEX-2TK (both Invitrogen, Karlsruhe, Germany). Purification was achieved using affinity chromatography on Glutathione Sepharose™ High Performance-columns (GE Healthcare, Freiburg, Germany), as previously described by von Holtum et al. [21]. Until use in the vaccination experiments, rMSP was stored at −80 °C in sterile-filtered 50 mM Tris buffer (pH 8.0).

### 4.2. Immunization Trials and Challenge Infection

Sixteen male, helminth-free, Holstein Friesian calves, aged three to four months, were used in the trial. They were divided into four weight-balanced groups of four calves each. Following an acclimatization period of seven days, cattle were immunized intramuscularly in the neck on SD 0, 21 and 42. The first group received 200 µg rMSP emulsified in 750 µg Quil A (Gerbu Biotechnik, Galberg, Germany) as adjuvant (rMSP Quil A group). The second group also received 200 µg rMSP but 400 μL Al(OH)_3_ (corresponding to 4 mg Al, Gerbu Biotechnik, Galberg, Germany) was used as adjuvant (rMSP Al(OH)_3_ group). The adjuvant-only control groups received the same amount of Quil A and Al(OH)_3_ as the vaccine groups, respectively, mixed with sterile-filtered 50 mM Tris buffer (pH 8.0) instead of the antigen. 

On SD 63, 64 and 65, calves were challenged with an oral infection dose of 1100 L3 each (total dose of 3300 L3 per calf, field isolate HannoverDv2000). During the course of the study, regular clinical examinations were carried out to record any local vaccination reactions and respiratory symptoms due to the challenge infections. Furthermore, all animals were weighed prior to first immunization and at the day of challenge infection, as well as at necropsy on SD 98. 

### 4.3. Determination of Larval Shedding

To ensure that no calf was infected before the scheduled challenge infection with *D. viviparus*, fecal samples were taken rectally three times before the start of the study, as well as on SD 0, 21, 42 and 63. Following challenge infection, fecal samples were taken daily from SD 84 onwards until necropsy to quantify larval shedding. Fecal samples (2 × 10 g) were analyzed using the Baermann technique, and the number of larvae per gram feces (LPG) was determined. LPG values from SD 84 to 98 were summed for each individual and geometric, as well as arithmetic, means and their standard deviations were calculated for each group, as described before [14]. 

### 4.4. Determination of Worm Burden and Worm Size

After necropsy on SD 98, lungs of individual cattle were removed. To retrieve adult worms, lungs were dissected, as described by Strube et al. [14]. The number of adult male and female worms was determined. In cases of incomplete worms, only the rear ends were counted and sexed. To estimate if the vaccine had an influence on adult worm size, 100 randomly selected female and 100 male worms per group (8–27 males/females per individual calf) were measured in length. For this purpose, worms were pinned on a black background and photographed with a ruler as scale. The digital images were analyzed by means of Olympus DP-Soft C-5050 software (Olympus Europa SE & Co. KG, Hamburg, Germany). 

### 4.5. Determination of Antibody Development 

Blood samples were taken once a week from SD 0 until necropsy at SD 98. Antibody development against vaccinated rMSP was determined by ELISAs detecting immunoglobulin classes IgM, IgA and whole IgG, as well as the subclasses IgG1 and IgG2.

Nunc Immobilizer™ Amino plates (Fisher Scientific, Schwerte, Germany) were coated with 0.5 μg rMSP dissolved in PBS by overnight incubation at 4 °C. Plates were washed three times using PBS containing 0.05% Tween (PBST) before 100 μL of sera (diluted 1:40 in PBS) was added to each well. After 1 h of incubation at 37 °C, the wells were washed three times with PBST. Subsequently, 100 μL/well of secondary antibodies diluted 1:10,000 in PBST was added, and plates were incubated for 1 h at 37 °C. Secondary antibodies were sheep anti-bovine IgA:horseradish peroxidase (HRP), sheep anti-bovine IgG:HRP, sheep anti-bovine IgG1:HRP, sheep anti-bovine IgG2:HRP and sheep anti-bovine IgM:HRP (AbD Serotec, Düsseldorf, Germany). After washing three times with PBST, wells were filled with 50 μL o-phenylenediamine hydrochloride (Sigma-Aldrich, Munich, Germany) dissolved in 25 mM citrate/50 mM phosphate buffer containing 0.04% hydrogen peroxide. After 10 min of incubation at room temperature in the dark, the reaction was stopped by addition of 50 μL/well 2.5 M sulfuric acid. Optical density (OD) was measured at a wavelength of 490 nm using the ELx800™ Absorbance Microplate Reader (BioTek, Bad Friedrichshall, Germany). All serum samples were analyzed as duplicates. For each set of duplicates, the mean was calculated for data analysis.

### 4.6. Binding of Anti-rMSP Antibodies to Native MSP

To test whether anti-rMSP antibodies of vaccinated animals bind to native MSP, immunoblot analysis was conducted using whole adult lungworm lysate as an antigen. Per lane, 16 µg crude antigen from female and male worms was separated by 12% SDS–PAGE followed by transfer to a nitrocellulose membrane. Immunoblotting was done using cattle sera from SD 0, 63 and 98 from the rMSP Quil A group, the rMSP Al(OH)_3_ group and the Quil A-only control group. Sera were diluted 1:20 in TBS containing 0.05% Tween-20 (TBS-Tween). Binding was visualized using a monoclonal anti-bovine IgG antibody conjugated to alkaline phosphatase (Sigma-Aldrich, Munich, Germany; diluted 1:30,000 in TBS-Tween) and BCIP/NBT substrate solution (Sigma-Aldrich). Sera from lungworm-infected and lungworm- negative animals were used as positive and negative control, respectively.

Furthermore, longitudinal sections of adult *D. viviparus* were analyzed by immunohistochemistry to show the localization of native MSP in male and female worms. Briefly, sections were deparaffinized with xylene and rehydrated in a graded alcohol series. Antigen retrieval was achieved by immersing the sections in citrate buffer (pH 6.0) at 70 °C overnight. For immunohistochemical staining, the Dako EnVision+ System-HRP (DAB) kit (Agilent Technologies, Santa Clara, CA, USA) was used according to the manufacturer’s instructions. A monoclonal anti-*C. elegans* MSP antibody (available under the designation 4A5 from the Developmental Studies Hybridoma Bank, Iowa City, IA, USA) diluted 1:10 in PBS-5% goat serum was used as primary antibody. Incubation time with the liquid DAB+ substrate-chromogen solution was 8 min for female lungworms and only a few seconds for male lungworms. Counter-staining was performed using Hematoxylin QS (Vector Laboratories, Burlingame, CA, USA) followed by dehydration using a graded alcohol series. 

### 4.7. Statistical Analyses

The sum of daily LPGs from SD 84 to 98, the number of adult worms, the number of larvae shed per female worm and mean worm length per animal, as well as weight gain between SD 0 and 98, were compared between vaccination and adjuvant-only control groups using Mann–Whitney U tests (SigmaStat software v. 3.1, Systat Software, Germany). A *p*-value ≤ 0.05 was considered statistically significant.

## Figures and Tables

**Table 1:** Geometric and arithmetic mean worm and larvae counts from rMSP-vaccinated and control animals.

	rMSP Quil A (N = 4)	Quil-A-Only (N = 4)	rMSP Al(OH)_3_ (N = 4)	Al(OH)_3_-Only (N = 4)
**Total worm count**				
GM	320.56	155.56	203.68	518.67
AM	407.50	188.75	348.50	563.50
**Female worm count**				
GM	202.61	93.38	121.87	302.79
AM	244.00	117.00	195.75	327.50
**Male worm count**				
GM	115.50	61.55	81.35	208.65
AM	163.50	71.75	152.75	
**Larvae/female worm**				
GM	15.04	32.44	30.31	21.07
AM	16.23	35.62	36.83	22.69
**Sum of daily LPG ^1^**				
GM	3029.81	3012.51	3653.46	6359.34
AM	3200.25	4272.75	4407.25	8179.00

^1^ sum of daily LPGs from study day 84 to 98. AM: arithmetic mean; GM: geometric mean; LPG: larvae per gram feces.

## Data Availability

The data supporting the conclusions are contained within the article.
